# Understanding Grammars through Diachronic Change

**DOI:** 10.3389/fpsyg.2017.01226

**Published:** 2017-07-31

**Authors:** Nerea Madariaga

**Affiliations:** Faculty of Arts, University of the Basque Country (UPV/EHU) Vitoria-Gasteiz, Spain

**Keywords:** syntactic change, Old Russian, Modern Russian, variation, object case marking, accusative case, genitive case

## Abstract

In this paper, I will vindicate the importance of syntactic change for the study of synchronic stages of natural languages, according to the following outline. First, I will analyze the relationship between the diachrony and synchrony of grammars, introducing some basic concepts: the notions of I-language/E-language, the role of Chomsky's (2005) three factors in language change, and some assumptions about language acquisition. I will briefly describe the different approaches to syntactic change adopted in generative accounts, as well as their assumptions and implications (Lightfoot, 1999, 2006; van Gelderen, 2004; Biberauer et al., 2010; Roberts, 2012). Finally, I will illustrate the convenience of introducing the diachronic dimension into the study of at least certain synchronic phenomena with the help of a practical example: variation in object case marking of several verbs in Modern Russian, namely, the verbs denoting avoidance and the verbs *slušat'sja* “obey” and *dožidat'sja* “expect,” which show two object case-marking patterns, genitive case in standard varieties and accusative case in colloquial varieties. To do so, I will review previous descriptive and/or functionalist accounts on this or equivalent phenomena (Jakobson, 1984 [1936]; Clancy, 2006; Nesset and Kuznetsova, 2015a,b). Then, I will present a formal—but just synchronic—account, applying Sigurðsson (2011) hypothesis on the expression of morphological case to this phenomenon. Finally, I will show that a formal account including the diachronic dimension is superior (i.e., more explanative) than purely synchronic accounts.

## Introduction

It seems a straightforward assumption to acknowledge diachronic change as the most important source of variation in languages and a crucial factor in shaping grammars. It is difficult not to agree with Lightfoot (in preparation) in that “nothing in syntax makes sense except in the light of change,” paraphrasing, in turn, the famous adagio by Dobzhansky (1973) that “nothing in biology makes sense except in the light of evolution.” Given the fact that most variable properties in languages emerge through change, it seems reasonable to include the relevant historical facts in any study on variation, at least in those cases when the history of the language concerned is sufficiently attested.

However, the role of historical linguistics does not receive the attention it deserves in synchronic studies. In this paper, I vindicate the importance of introducing the diachronic dimension into the formal study of at least certain synchronic phenomena, by highlighting the role of syntactic change through a specific example of variation in Russian. First, I analyze the relationship between diachrony and synchrony of grammars, introducing some basic concepts: the notion of syntactic change, its abruptness and discreteness, the contrast between I-language and E-language, the relevance of language acquisition, and its role in syntactic change, as well as the effect of Chomsky's (2005) third factor in language change. Further, I describe the case alternation between genitive vs. accusative complements of certain medial verbs in present-day Russian (the so-called –*sja* verbs). Then, I review the shortcomings of purely synchronic accounts of different linguistic orientations applied to this specific case of variation. Finally, I prove that an account introducing the diachronic dimension can be explanatorily superior, at least, in this specific case study on variation. The final section contains some conclusions to this paper.

## Basic notions about diachronic generative syntax

In this section, I will introduce some basic notions on historical change assumed by generative approaches to grammar (as opposed to other linguistic schools, mainly usage-based or functionalist approaches). Diachronic generative studies started in the early 70s, with Andersen's (1973) article on abductive change and Lightfoot's (1974) work on modals, preceded by Klima's (1965) dissertation (*Studies in diachronic transformational syntax*). The foundational work on diachronic generative syntax is unanimously considered to be Lightfoot's (1979) *Principles of diachronic syntax*; it gave rise to a productive research program within formal linguistic studies. As an example, see the collective volumes, the product of the biennial DIGS (*Diachronic Generative Syntax*) conference, published by OUP. Recently, CUP published the collective reference handbook on diachronic generative syntax *Cambridge handbook of historical syntax*, edited by Ledgeway and Roberts (2017).

A basic notion in generative approaches to diachrony is the view of syntactic change as a special kind of “reanalysis” or rather “new analysis,” as firstly claimed by Lightfoot (1979 and subsequent work—1991, 1999, 2006), and later widely adopted in the generative linguistic community (Faarlund, 1990; Hale, 1998; Roberts and Roussou, 2003; van Gelderen, 2004; Roberts, 2007; etc.). Within this view, learners acquire a language by parsing or analyzing the relevant input, also called Primary Linguistic Data (PLD). Most of the time learners succeed in converging with the grammar/structure that generated its input, a property called *inertia* in formal grammar (Longobardi, 2001). Syntactic change, then, stems from a special type of “analysis” or “parsing” of the PLD a learner can perform during the language acquisition process; namely, in the case when, for some reason, the learner's grammar/structure does not converge with the grammar/structure that generated its input. This is known as the discontinuity or failure of transmission between generations.

In generative approaches to change, the discontinuity of transmission is usually assumed to be abrupt (rather than gradual), in the sense that grammars are acquired afresh by each speaker (Lightfoot, 1979, 1991 and afterward). What seems like gradual change is reduced in diachronic generative syntax to successive discrete changes according to the following considerations: (i) A change can initially affect only specific items or structures, and then spread to more items or syntactic environments (van Gelderen, 2010; Madariaga, 2012). (ii) A change can spread through a linguistic community, giving rise to situations of diglossia and “competing grammars” (Kroch, 1989; Yang, 2002). (iii) A change can produce different synchronic variants coexisting in a single speaker at different linguistic levels, which we commonly call “I-language” vs. “E-language.” I-language stands for the linguistic competence of each individual speaker, while E-language refers to the linguistic productions of a community of speakers (Chomsky, 1986, p. 7–8). This distinction has proven very useful to discriminate internal properties of grammars and linguistic features, dependent on external sociolinguistic considerations (Sobin, 1997; Lightfoot, 1999; Lasnik and Sobin, 2000; Madariaga, 2009; etc.).

Among formalists, there is common agreement in that linguistic change is contingent, in the sense that the initial trigger of a shift in grammar is primarily originated in language performance/E-language, which is partly determined by extra-linguistic factors and can change in unpredictable ways (Faarlund, 1990; Lightfoot, 1991, 1999, 2006; Roberts, 2007). The conditions of language transmission can be altered by modifications of the PLD, triggered by external random sociolinguistic factors, phonological erosion, previous unrelated morphosyntactic changes, drops in frequency of the relevant input, etc.

Some authors, however, refine this idea by proposing certain regularities imposed by our Language Acquisition Device (LAD), which can lead learners to acquire a structure in a new way with respect to the previous generation of speakers, thus giving rise to diachronic change. This is depicted by some authors in the form of hierarchies arranging the parametric choices available in acquisition according to more or less marked options. These options determine the probability of a parameter to be set in one way or another and, therefore, the possible ways in which change will most likely take place (Roberts, 2007, p. 267ff, 2012; Biberauer et al., 2010).

Other biases determining, at least partially, language change are considerations of optimality, economy, and a tendency of grammars to become simpler (van Gelderen, 2004). This is in line with Chomsky's (2005) “third factor,” which can be defined as those language independent principles of structural architecture, efficiency, and processing that render language as an optimal solution to the interface (phonological and semantic) conditions.

According to these previous notions, the fundamental role of diachronic change as a “language shaper” is then two-fold, as it can affect internal grammars (I-language) or just remain at a “surface” level, modifying the speakers' external productions in E-languages. Here are the views at this respect:

- Diachronic changes affecting I-languages are in the first place related to language acquisition, Chomsky's (2005) “second factor.” As we said before, formal approaches to diachrony assume that change takes place between two different generations of speakers during the language acquisition process (cf. an illustrative case study in Duguine and Irurtzun, 2014). With the advent of the minimalist program, third factor effects are also acknowledged to be implied in diachronic change (Biberauer and Roberts, 2016). Some examples are the Minimax thesis (Chomsky, 2009; Fodor, 2009), according to which parameters must be understood as an optimal solution to the conflict between UG and learnability (“minimize genetic information and optimize the amount of learning”), the role of Feature Economy in grammaticalization processes (van Gelderen, 2004), and the spread of a specific change through different structures or lexical items by Input Generalization (Roberts, 2007; “generalization of the input”).
- Diachronic changes affecting E-languages, i.e., understood as innovations at an adult age (cf. the concept of “emergent grammars,” as in Hopper, 1987) are mostly disregarded in formal accounts. However, as said before, in the “contingent” type of diachronic generative approaches (Lightfoot, 1991, ff), this kind of innovative or surface modifications of the PLD are acknowledged as potential initial triggers for further changes in I-grammars.

In this respect, a third area must be considered, namely, Externalization processes (Chomsky, 2010; Sigurðsson, 2011), as we usually call the ways of mapping I-features into more external components, e.g., the morphological realization of abstract syntactic features, which will be the central topic in this paper.

All these considerations lead us to ask ourselves about the locus of variation in minimalism. Here we also face different options, which do not necessarily exclude each other: (i) an older idea is the so-called Borer (1984)-Chomsky (2001) Conjecture, that all variation is contained in the Lexicon; (ii) a later refinement of this idea is to admit that the interfaces themselves, in addition to the Lexicon, can also answer for linguistic variation (e.g., Biberauer, 2008, p. 32); and (iii) finally, we observe additional third-factor clustering effects across languages, probably related to the specific ways of mapping syntactic structures into the interfaces (Biberauer et al., 2010; Boeckx, 2011; Roberts, 2012).

In what follows, I will focus on the main goal of this paper, which is to vindicate the role of historical change in formal accounts. This idea does not imply that change has a direct effect on synchronic stages of a language, because we know that speakers do not have access to the I-grammars of previous generations (as represented in Andersen's, 1973 Abduction principle). But diachronic change definitely can shed light on the ways variation has to be understood, and even on the paths that I-languages follow in order to be configured.

Diachrony interacts with synchronic accounts in different ways, for example, a fundamental reason that led some scholars to revisit cartographic and lexicalist approaches to the synchrony of languages was the need to explain acquisition and change through it (Roberts, 2012). But the study of historical change also helps us understand synchronic language-specific properties and concrete instances of variation (cf. the examples in Lightfoot (in preparation), or even at a methodological level, it can help us decide between two alternative explanations of a synchronic phenomenon (see e.g., Ormazabal and Romero, in preparation).

Following these lines, the case study presented in the following sections constitutes an illustrative example of how diachronic data can clarify the puzzle posited by an instance of variation in a synchronic stage of a language.

## A synchronic variation phenomenon: case alternation in russian –*sja* verb objects

In this section, I provide a synchronic description of our case study. I will focus on the phenomenon of case alternation between genitive and accusative case marking on the object of some medial verbs in Russian, which are virtually all the –*sja* verbs expressing fear or avoidance, as well as the verbs *slušat'sja* “to obey” and *dožidat'sja* “to expect.” These verbs display genitive object case marking in standard varieties (1) and accusative case marking in colloquial varieties (2); cf. Peškovskij (2001 [1938], p. 278) and Krys'ko (1997).

(1) a.  On         boitsja *ženy.*                 (Standard variant)
he.nom fears    wife.gen
“He is afraid of his wife.”
b.  Devočka vsegda slušaetsja *materi*.
girl.nom always  obeys      mother.gen
“The girl always obeys her mother.”
c.  Inspektor doždalsja *kollegi*.
inspector waited     colleague.gen
“The inspector waited for his colleague.”
(2) a.  On         boitsja *ženu*.                  (Colloquial variant)
he.nom fears    wife.acc
“He is afraid of his wife.”
b.  Devočka vsegda slušaetsja *mamu*.
girl.nom always  obeys      mum.acc
“The girl always obeys her mom.”
c.  Paren’        doždalsja *devušku* iz      armii.
young man waited     girl.acc   from army
“The young man waited for his girlfriend from her
                  military service.”

The verbs implied in this alternating pattern are the following (*ap*. Peškovskij, 2001 [1938], p. 278): (i) all the –*sja* verbs of fear, avoidance, separation: *bojat'sja* “to be afraid,” *storonit'sja* “to avoid,” *pugat'sja* “to be frightened,” *stydit'sja* “to be ashamed,” *osteregat'sja* “to beware of,” *opasat'sja* “to be afraid, to mistrust,” *strašit'sja* “to dread,” č*uždat'sja* “to keep oneself aloof,” *lišat'sja* “to be deprived,” *stydit'sja* “to be ashamed,” *konfuzit'sja* “to feel ill at ease,” *stesnjat'sja* “to be timid,” etc.; (ii) the weak intensional –*sja* verbs *slušat'sja* “to obey,” and *dožidat'sja* “wait, expect” (the only representatives of this kind nowadays).

Timberlake (2004, p. 317) offers a semantic classification of the Russian verbs taking lexical genitive case nowadays (cf. also (Kagan, 2013), for a more detailed semantic account). Those are verbs including the following semantic components^1^:

- “Potential,” i.e., “contact is potential but unrealized,” the so-called weak intensional verbs (*iskat*' “search for,” ž*da*t' “await,” *trebovat*' “demand,” ž*elat*' “desire,” *xotet*' “want”). Almost all of them are active (without the suffix –*sja*), and their alternation pattern is semantically determined, unlike the alternation discussed in this paper. Only the –*sja* forms in this group, *slušat'sja* “obey” and *dožidat'sja* “wait,” follow the pattern (1–2) addressed here, and do not share the semantically determined alternation of their active counterparts, as we will see in the last two sections of this paper.
- “Avoidance,” whose semantics is described as “possible contact is avoided,” a notion conveyed by the verbs of fear and separation analyzed here.
- “Tenuous,” defined by Timberlake as “actual contact in the face of possible non-contact” (*dobivat'sja* “achieve, acquire,” *kasat'sja* “touch on”). For the purpose of this paper, I will classify these verbs together with the weak intensional potential verbs, because they share the semantic feature of “potentiality,” and behave in the same way historically.

In this paper, I will leave aside the active weak intensional verbs of the type *iskat'* “search,” ž*dat'* “wait,” *trebovat'* “demand” (type i) because the distribution of the variants in them is radically different to the one discussed here. Weak intensional active verbs, unlike the verbs under study in this paper, display a clear cut semantic distribution of case marking: roughly, genitive case is used with potential but unreal/“unbounded” objects (usually abstract nouns, but also some concrete but indefinite objects), as in (3a); and accusative case for concrete or real/“bounded” objects, definite or not, as illustrated in (3b) (see Timberlake, 2004; Kagan, 2013).

(3) a.  My ždëm otveta         / My trebujem vašego vnimanija.
we wait   answer.gen / we demand   [your attention].gen
“We are waiting for an/the answer/We demand your attention”
b. My ždëm žurnal       / My trebujem naše bljudo.
we wait journal.acc / we demand [our dish].acc
“We are waiting for a/ the journal/ We demand our dish.”

The alternations in (3) imply a semantic contrast between genitive and accusative case marking, which is totally absent in the case of the verbs of fear/avoidance, or *slušat'sja* “obey” and *dožidat'sja* “expect,” illustrated in (1) vs. (2). In the latter, much fuzzier factors dealing with declension class, language level, and the speaker's age are involved, as we will see later on in this paper^2^. The nature of this alternation strongly suggests that we are dealing with a change in progress:

First, there is an undoubtedly high degree not only of interspeaker variation, but also of intraspeaker variation, which points to a situation of double coding or, at least, of competing grammars (Kroch, 1989; Yang, 2002), introduced in the previous section.

Second, some authors have observed an increase of the accusative pattern in recent decades, together with a higher frequency of use of the accusative pattern among younger speakers and colloquial registers (Krys'ko, 1997; Nesset and Kuznetsova, 2015a).

Finally, this alternation displays the typical “peripheral” properties of certain linguistic phenomena (as described in Baker, 1991; Sobin, 1997; Lasnik and Sobin, 2000; Madariaga, 2009; cf. the distinction between I-level vs. E-level phenomena in the previous section):

- Inconsistent or contradictory productions and intuitions about the variants;
- In many cases, free optionality of choice between two variants;
- Non-natural late acquisition of one of the variants (learning based on repetition, rules taught at school), or frequent exposition/priming^3^;
- The more frequent or colloquial the lexical item at issue, the more often accusative case is used; for example, the occurrences of the accusative variant outnumber those in the genitive case if we perform a simple Google search for the combination *bojat'sja* “be afraid” and *mama* “mom” (colloquial variant), while the percentages differ when we search for the same verb + *mat'* “mother,” a less colloquial lexical item, as shown in (4) vs. (5)^4^:

(4) a.  bojat’sja mamu.ACC     58.8% of occurrences
b.  bojat’sja mamy.GEN     41.2% of occurrences
(5) a.  bojat’sja mat’.ACC       25% ofoccurrences
b.  bojat’sja materi.GEN     75% of occurrences
“Be afraid of one’s mother”

In the following section, I will apply different approaches and hypotheses (non-formal and formal) to this phenomenon of variation, and review their advantages and shortcomings. Afterward, I will offer my own proposal, which introduces diachronic data, and show in which way it is more explanatory than the purely synchronic accounts proposed so far.

## Synchronic approaches according to different linguistic orientations

### Non-formal approaches

In this section, I will review three previous studies on this specific topic or in more general, but directly related, phenomena of the Russian language. All these studies have been performed from the perspective of structuralist or functionalist approaches. Albeit there are noticeable differences between them, these approaches appear as just descriptive and, in some cases, also incomplete.

#### Decomposition of grammatical case

Scholars of structural linguistic orientation explored the possibility of decomposing grammatical case into smaller semantic features. Each grammatical case would be in this way characterized by a group of features that enter into syntagmatic and paradigmatic relations. The presence of common features among different cases would allow for replacing one case with another when they share most features, or extending the uses of a case by addition or loss of the relevant features.

One of the most renowned examples of this system is precisely decomposition of Russian case, proposed by Jakobson (1984 [1936]), and revised later by Franks (1995). These authors do not specifically address the alternation in case marking under study in this paper, but they do examine a related morphological syncretism, namely, the conflation of genitive and accusative morphological cases on animate objects (masculine singular and all plural). This conflation is illustrated in (6b):

(6) a.  Vasilij  Ivanovič xorošo znaet   *moj gorod.*
Vasili  Ivanovich well      knows [my town].nom/acc
“Vasili Ivanovich knows my town very well.”
(cf. *Èto moj gorod*.nom “This is my town”)
b. Nikolaj  Borisovič  xorošo znaet    *moego zjatja.*
Nikolai Borisovich well      knows [my      son-in-law].
                                                                    gen/acc
“Nikolai Borisovich knows my son-in-law very well.”
(cf. *Èto rabota moego zjatja*.gen “This is my son-in-law’s
                                                                               work.”)

According to these authors' system of case decomposition, genitive case would consist of the features [+oblique, –marginal, –non-ascriptive], while accusative would be defined as [–oblique, –marginal, –non-ascriptive]. In order to obtain (6b), they just erase the distinction between the two forms by the feature [oblique] in an operation that equals both forms with the characterization [–non-ascriptive, –marginal], and renders the morphological syncretism we observe in the language at the synchronic level.

Hypothetically applying the same system at the diachronic level, we could also claim that accusative and genitive morphological cases can alternate by erasing their [oblique] feature in the relevant context, leaving both forms with equal features ([–non-ascriptive, –marginal]). This operation would render the alternation between genitive and accusative in the complements of the verbs discussed in this paper.

This proposal is very appealing, at least, if we settle for a basic morphological description. Nonetheless, the mechanism of decomposition of grammatical cases—and related morphological operations—is just descriptive, and maybe not so persuasive on the basis of independent evidence. At times, the correspondence of a case to the alleged underlying semantic features is not very informative; for instance, in the specific alternation under study here, the characterization of the genitive and accusative cases does not capture the semantic values of avoidance and potentiality, which clearly differ from the usual values of these two cases in other parts of the grammar.

#### Maps of semantic notions

Another system inspired by the theory of case decomposition is the more recent idea of representing the semantic values underlying grammatical cases with the help of the so-called “maps of semantic notions.” These maps include the various semantic interpretations of the cases existing in a language or a group(s) of languages, and depict the higher or lower plausibility of syncretism or transfer between cases through the representation of the “geographic” distance between the different values.

The closest study to the phenomenon discussed in this paper is to be found in Clancy (2006), based on Haspelmath (1997). He offers a topology of Slavic case with multidimensional scaling, in which the distance between different functions or semantic values intends to capture frequencies of use, markedness of the variants, and possible changes and syncretisms. Thus, broadening or restricting the “meanings” or semantic values of a specific case should correspond to contiguous or related areas on the semantic map.

Clancy (2006, p. 24) captures the relationships between the semantic values of Slavic case in such a map of semantic notions, depicting the “distance” between those values. Such an approach is interesting from the point of view of case morphology, and in this specific study, it is very detailed. However, as it stands, it is of no use for our analysis, as the semantic notions on Clancy's map associated with the alternating cases addressed here (“dist from/afraid of,” which stands for the ablative value of the genitive case, including verbs of fear, and “understand,” which stands for regular direct objects) are too far away from each other to accommodate our variants and hypothesize a possible transfer between them. This can be due to the fact that Clancy (2006, p. 25) himself acknowledges that the dataset used for this specific map was a pilot one and thus incomplete, but in any case, such a representation of the semantic values of case is purely descriptive and does not explain the reason for the variation phenomenon under study.

#### Paradigms or construction networks

Construction networks can be defined as a descriptive tool developed within the Construction Grammar, a functionalist approach to languages. Within this approach, Nesset and Kuznetsova (2015a,b) have addressed the specific phenomenon discussed in this paper. More specifically, the authors count the occurrences of the accusative and genitive variants associated with several –*sja* verbs in the Russian National Corpus and aim to account for the asymmetries in the use of these alternating variants.

As introduced in the previous section, they find differences depending on the register (whether the utterance is part of a corpus or a spontaneous production), the age of the speaker (accusative more frequent in younger speakers), and the specific lexical item. Interestingly, the verbs more often found in combination with an accusative complement are, according to their search in the National Corpus, the verbs *slušat'sja* “to obey,” *bojat'sja* “to be afraid,” *dožidat'sja* “to wait,” *dostigat'* “to reach,” and *izbegat'* “to avoid;” there are also differences among them, namely, the verb *slušat'sja* and after it, *dožidat'sja*, are much more frequently combined with accusative than the rest (Nesset and Kuznetsova, 2015a, p. 371–373). We will come back to this fact in the following section.

The main hypothesis in their papers is that a high level of individuation or animacy favors accusative case marking, as happens in other cases of alternation between genitive and accusative case (namely, the syncretism of animate NPs in object position, to which we will return later on). All other factors (declension class of the noun, the intensional or directional semantics of the verb, the “opacity” of the –*sja* suffix) are relegated by them as epiphenomenal.

After describing the conditions for case alternation, Nesset and Kuznetsova accommodate all the relevant variables in a paradigm or construction network, which can be defined as the representation of a specific construction and of some of its subtypes, together with the relevant features, such as markedness of choices, statistical significance, possible diachronic changes, variation, etc. Nesset and Kuznetsova (2015a, p. 388) provide such a construction network for three of the verbs involved in the alternation at issue. But, again, the statistics offered by these authors, as well as the intervening factors, are very illustrative of what is happening in the language, but their network is just descriptive. Another shortcoming of the account is that it overlooks the potential syntactic motivations behind the variants, which will play a fundamental role in the alternation, as we will see in the two final sections.

A final observation regards the imprecise semantic and syntactic characterization of the verbs these authors analyze. If we follow Timberlake's (2004, p. 317) classification of the Russian verbs taking lexical genitive nowadays (cf. previous section), *slušat'sja*, and *dožidat'sja* are weak intensional verbs (the only “potential” –*sja* verbs), *bojat'sja* and *izbegat'* are verbs of avoidance (one medial, the other active), and *dostigat'* is a potential verb with active form. The indistinct treatment of all these forms leads the authors to lump together verbs of different syntactic and diachronic behavior.

### Formal synchronic approaches

To the best of my knowledge, there are no formal studies on this specific alternation phenomenon of the Russian language, so in this section I will try to apply more general purely synchronic formal accounts to it.

As a first step, we could just think that the alternation discussed here is not relevant for syntax, i.e., that it arose due to a spontaneous change in the relevant morphological rule instruction; the rule formerly realizing genitive case on the object of these verbs would have just been modified by a new rule specifying that these objects must be accusative.

Of course, this can be true from a strict synchronic point of view, but still some questions remain unanswered: (i) Why does this alternation exist? (ii) Why does it match the distinguishing features of a change in progress? (iii) Why is this alternation not uniform (depending on animacy, declension classes, the presence of –*sja*, etc.). We will answer these questions in the following section.

In a more refined way, we could try to apply to this alternation Sigurðsson (2012) system of regular vs. quirky morphological cases in Icelandic. According to Sigurðsson (2012), the expression of m(orphological)-case corresponds to the Externalization component, i.e., to the different ways of assignment or realization of PF-exponents with respect to underlying syntactic features. Crucially, his system acknowledges the presence of “third factor” properties, namely, markedness of the PF-exponents. In other words, some morphological markers are more or less eligible to encode what is located in the corresponding syntactic heads.

Applying these insights to our alternation in (1–2), partially repeated below for convenience, accusative case in (7b) would be an unmarked variant, while genitive case in (7a) would correspond to a marked (quirky) variant in this specific configuration.

(7) a. On        boitsja *ženy.*               (Standard variant)
he.nom fears   wife.gen
“He is afraid of his wife.”
b. On        boitsja *ženu.*               (Colloquial variant)
he.nom fears   wife.acc
“He is afraid of his wife.”

Formalizing this observation, we obtain a characterization of the alternating variants and the shift between them: the marked genitive quirky case variant is represented in (8a), while (8b) stands for unmarked accusative case. The change from genitive into accusative in the relevant contexts would be as in (8c), from marked into unmarked:

(8) a.  Genitive direct objects: v^*++^
b.  Accusative direct objects: v^*^
c.  Change: v^*++^ > v^*^

This is undoubtedly so at a strict observational level but, as in the previous accounts, it is just descriptive. Sigurðsson (2012) himself acknowledges his system as descriptive, because, he says, it is the only thing we can do when dealing with the Externalization component of the language.

In the rest of the paper, however, I will argue that a more precise (though still formal) account is possible if we pay attention to the diachronic dimension of a phenomenon. More specifically, I will show that what seems like a m-case alternation between genitive and accusative case marking hides in fact two different structures inherited from a quite complex historical shift that took place in the history of the Russian language several centuries ago.

## A formal diachronic–synchronic analysis

### The decline of bare lexical genitive case in early russian

In this section, I will argue for the proposal that the alternation between accusative and genitive case marking related to –*sja* verbs did not originate in a spontaneous change in markedness of the m-cases involved. As an alternative, I will propose that it is, in fact, the last step in a long-term change associated with a global reorganization of case marking in Russian.

First, I will show that the alternation in -*sja* verbs, illustrated in (1–2), is not new to the language, but is rather the replication of a prior change from genitive into accusative object case marking, which had taken place in Middle Russian in active verbs. We will see that we are then dealing with a unique change happening at different moments under distinct structural conditions.

In early Indo-European languages, “bare” grammatical cases (i.e., lacking any overt adposition) were often used as lexical case markers of NPs in a variety of syntactic functions and with diverse semantic values. Later on, we observe a tendency to replace bare case endings by adpositional phrases depending on the language or group of languages (Bauer, 1995; Hewson and Bubenik, 2006)^5^.

This was precisely the case of early Slavic and early Russian. Here, bare cases were regularly used in non-structural positions, namely, encoding “oblique” NPs (adjuncts), and also complements of lexical heads. The examples in (9) illustrate different adjuncts marked with bare lexical cases, alternating already in early Slavic with overt prepositions (Borkovskij, 1978, p. 364ff).

(9) a. Otstupi voleju *Kyeva.  (1^st^ Novgorod Chronicle,* 36)
left       by-will Kiev.gen
“He moved away from Kiev by his own free will.”
b. Inii    mnozi nesoša i     *Volodimerju*  a     otudu
other many carried him Vladimir.dat and from-there
*Kyjevu.*                               *(Laurentian Chronicle, 69)*
Kiev.dat
“Some of them carried him to the town Vladimir, and from
there, to Kiev.”
c.  Izjaslav sěde    *Kyevě,    *Svjatoslavъ *Černigově*.
Iziaslav  settled Kiev.loc Sviatoslav   Chernigov.loc
(*Laurentian Chronicle,* 55)
“Iziaslav settled down in Kiev, and Sviatoslav in Chernigov.”

Bare cases, including genitive case, could also encode quirky objects of various types in a regular and much broader way than today.

(10) a.  I    *vsego*   na nemo pytati i      *bezčinija*       i
and all.gen to  him   ask     and inactivity.gen and
nevěžestva.
ignorance.gen     *(House-Orderer,* 36)
“He must respond for all, for the things that have not been
done and those that he does not know.”
b.  Zaby   *inočeskogo obeščanija*.
forgot [of-monk     promise].gen
*(Life of Dimitri,* 210b, in Borkovskij, 1978: 353)
“He ignored the monastic vow.”
c.  I          *vsjago togo zapasu*                 ključniku
and      [all       this   provision].gen      housekeeper
vĕdati.
administrate.           *(House-Orderer,* 54)
“And the housekeeper must take care of all these
supplies.”

By that time, the old Slavic case system was undergoing a major reorganization. Bare lexical cases were (i) either replaced by overt PPs (adjuncts), as shown in (11) below, (ii) either reinterpreted as non-lexical or structural cases, (iii) or, some times in the case of dative and genitive cases, lost altogether and replaced with accusative case, as we will see soon.

The replacement of bare lexical adjuncts by PPs was completed by late Old Russian—early Middle Russian. The examples in (11) correspond to a later copy of the same texts from which examples in (9) have been extracted^6^. The only difference between them is the addition of overt prepositions in the case of the later copies:

(11) a.  Otstupi   voleju   *is*       Kyeva.
left          by-will from   Kiev.gen
(1^st^ *Novgorod Chronicle—Commission roll,* 112b)
“He moved away from Kiev of his own free will.”
b.  Inii    otroci    nesoša    i    *k  Volodimerju*    a
ottudě        *k* Kyjevu.
other fellows carried    him to Vladimir.dat and
from-there to Kiev.dat
                                                   *(Radziwill Chronicle, 69)*
“Some comrades carried him to Vladimir, and from there,
                                                                         to Kiev.”
c.  Izjaslav sěde   *v  Kyevě*,      Svjatoslavъ *v* Chernigov.loc
Iziaslav settled in Kiev .loc Sviatoslavъ in Černigově.
                                                  *(Radziwill Chronicle, 55)*
“Iziaslav settled down in Kiev, and Sviatoslav in Chernigov.”

The replacement of bare genitive lexical case with PPs, together with its reanalysis as a non-lexical case, severely restricted the interpretation of the remaining bare genitive NPs as quirkies. The presence of such forms created a “disturbing” piece of evidence in the PLD that learners of Russian received, and tended to be progressively driven out from the language.

At this point, we can already realize the deep historical roots of the synchronic alternation addressed in this paper (1–2). In the following pages, I will show that this alternation was a distant product of this initial reorganization of the Old Russian bare case system. As such, it was ultimately tied to the more general typological change that took place in most early Indo-European languages; namely, a shift from Proto-Indo-European OV into VO word order, i.e., from left-branching into right-branching (Lehmann, 1974; Friedrich, 1975; Watkins, 1976; Luraghi, 1990; Bauer, 1995; cf. discussion in Keydana, in preparation, and Pancheva, 2008 for early Slavic). One of the consequences of this recurrent process in Indo-European implied precisely the replacement of bare lexical cases by PPs headed by overt prepositions (Lehmann, 1993; Bauer, 1995; Hewson and Bubenik, 2006). This is precisely the phenomenon observed in early Slavic, too, as illustrated in (9) vs. (11).

In the rest of this section, I will review the changes related to this general diachronic process, which preceded the alternation in case marking in (1–2). As noted before, this process proceeds in two steps of similar characteristics, but distant in time from each other. First, I will focus on the first step, the change from genitive into accusative case marking associated with regular active verbs, then I will account for the second part of this shift, that affected medial (–*sja*) verbs, and explain why it took place much later than the previous one.

### First step of the change: genitive into accusative complements of active verbs

#### The loss of bare genitive complements of active verbs

Bare genitive case associated with some active verbs was lost as soon as in prehistoric Slavic; other active verbs still display genitive object case in early Slavic. This shift affected several classes of active verbs, including the verbs discussed here, i.e., verbs traditionally classified in Indo-European linguistics as verbs of “separation” (avoidance) and verbs of “desire/achievement and perception” (potential).

##### Verbs of separation (avoidance)

They can denote physical or psychological avoidance. Savčenko (2003 [1974]) includes in the first group the Indo-European verbs expressing departure, typically associated with an ablative case that, in the languages with ablative-genitive syncretism, is expressed with genitive case (Greek and Balto-Slavic, including Old Russian cf. 12):

(12) Se  azъ otxožju *světa sego.*
this I     leave   [world this].gen
        *(Laurentian Chronicle* 54b)
“Now I am leaving this world.”

Some verbs maintained this pattern as an archaism until the nineteenth century in Russian, as shown in (13). But their complements were in general reinterpreted as adjuncts quite early, by adding an overt P such as *iz, ot, c* “from” (see example 11a above).

(13) Nadobno každomu bežat’ *ètogo Peterburga.*
need        each       escape [this  Petersburg].gen
                                              (Pisemskij, *Tycjača duš)*
“Everyone needs to escape from this Petersburg.”
(Nowadays: [_PP_ *iz* ètogo Peterburga] “from this
                                                    Petersburg”)

Psychological avoidance reflects a metaphoric sense of separation, and corresponds to the psych verbs denoting fear (Schmalstieg, 1983; Šaxmatov, 2001 [1941]). Some of them were active and displayed (ablative-)genitive case assignment in certain Indo-European languages, including early Slavic:

(14) Jego  *imene*      trepetaxu vsja strany.
his    name.gen feared     all    countries
                         (*Laurentian Chronicle*, 97b)
“All the peoples feared his name.”

In Middle Russian, the active verbs denoting fear lost genitive complements and replaced them by overt PPs, nowadays *pered* “before” + NP with instrumental case. This is now the pattern of *robet'* “to hang back,” *trusit'* “to fear, to be in a funk,” *trepetat'* “to tremble, to be afraid,” *drožat'* “to tremble,” etc. (15a). Gorbačevič (1971) reports the last literary archaic uses of bare genitive with active verbs in the nineteenth century (15b)^7^

(15) a.  Oni  drožat    *pered  Bogom*
They tremble before God
        (Griboedov, in Peškovskij, 2001 [1938], p. 278)
b. Odna liš’   ja *ljubvi*    do smerti trušu.
one    only I  love.gen to death  fear
“I am the only one who dreads love.”

##### Desire and achievement verbs and verbs of perception (potential)

These are verbs denoting “to want,” “to search for,” “to wait,” “to achieve,” and “to hear,” “to see,” “to feel,” etc. These active verbs alternated in early Indo-European languages between genitive and accusative case marking, and most of them changed later into an accusative or PP pattern (Savčenko, 2003 [1974]). This is the case of Old Russian, in which the following active verbs of perception are reported to have displayed an alternating pattern (Borkovskij, 1978, p. 346–347): č*itati* “to read,” *s*ъ*motriti* “to look,” *slyšati* “to hear,” *slušati* “to listen/to obey,” *viděti* “to see,” *očjutiti* “to feel.”

(16)  a.  I     *knižnago poučen*Ь*ja*    slušaita.
and  [bookish teaching].gen listen
                        *(Laurentian Chronicle, 151b)*
  “And you both listen to the teaching of the Bible.”
b. Ašče   kto   o(t)ca         li  *   m(a)t(e)re   * ne poslušaetЬ.
if       who father.gen or    mother.gen   not  hears
*                                   (Laurentian Chronicle,* 18b) “
“If somebody does not obey his father or mother.”

According to Borkovskij (1978, p. 347), such genitive objects stopped being available in Middle Russian (except for stylistically marked archaisms, which survived much longer in the language), and the verbs became regular transitive verbs taking an accusative object. This shift in case marking is illustrated in example (17), extracted from the late fifteenth-century copy of (16b):

(17)    Ašče                   kto     o(t)ca                 i      *m(a)t(e)rь*
if                         who   father.gen/acc  and  mother.acc
ne poslušaetь.                    *(Radziwill Chronicle,* 18b)
not hears
“If somebody does not obey his father and mother.”

Together with the verbs of perception, Borkovskij and Kuznecov (2004 [1963], p. 428) classify as genitive object verbs also the verbs denoting desire and achievement; almost all of them were active: *dobyvati* “achieve,” *iskati* “seek,” ž*dati* “wait,” *prositi* “ask,” *xotěti* “want,” etc.

(18)   a.  Uvčdavъ       Onanjьa,   xotja emu    *dobra.*
having seen  Onanya     wanted him  good.gen
                        *(1^st^ Novgorod Chronicle,* 134)
“He saw Onanya and wanted to help him
(lit. wanted the good for him).”
*(Prayer of Danilo Zatochnik*,in
Borkovskij and Kuznecov, 2004 [1963], p. 428)
b. Zane muži *zlata* dobudutъ.
because men gold.gen achieve
“Because men will make money.”

All these verbs are classified nowadays as weak intensional verbs and their historical development was different from the verbs of perception. As noted before, nowadays these verbs maintain the genitive vs. accusative alternation in objects but, unlike the alternation addressed in this paper, it is semantically determined (real/bounded vs. unreal/unbounded feature; cf. examples in (3) above).

##### Other verbs

Other Indo-European genitive objects of active verbs, which are relevant for Slavic, are reported in Savčenko (2003 [1974]) and Borkovskij (1978) to have been later reinterpreted as adjuncts (with instrumental case or PP), most of them already in prehistoric times. This was the case of the verbs denoting governing (“to govern,” “to rule”), verbs of “held part” (“to grasp,” “to hold by”), as well as speech verbs (“to say,” “to think,” “to remember”). Others changed into regular accusative objects, with the verbs meaning “taking care,” and sorrow (“to regret,” “to feel sorry”).

#### The rise of new alternations between the genitive and accusative cases in non-lexical positions

In parallel to the loss of bare lexical genitive case, we observe in Middle Russian a significant development of the genitive form as a non-lexical case, which became either reinforced in structural positions previously existing in the language, or spread to new syntactic positions.

The structural positions undergoing the genitive/accusative case alternation are the following: (i) regular animate objects, and (ii) NPs governed by some quantificational or negative head. Let us see some examples of them.

(i) The alternation in regular objects arose in Russian with the extension of the genitive case marker to animate regular objects of the masculine singular declension (*o*-stems) and plural declension (all stems). The process of replacement of the old nominative-accusative form by a genitive form in the relevant animate objects started already in Old Church Slavonic (OCS) (19) and was completed in Early Middle Russian (Krys'ko, 1994). Inanimate objects belonging to these stems remained marked with nominative-accusative case (20):

(19) a.  Ce    privěsę *č(e)l(ově)kŭ němŭ* běsenŭ.
here carried [person mute        possesed].nom(/acc)
    (OCS: *Codex Marianus* & *Zographensis,* Mt. 9:32)
“They brought him a mute man who was demon-possessed.”
b. Privedosę    emu    *č(e)l(ově)ka gluxa.*
carried        him    person            deaf.gen(/acc)
(OCS: *Liber Sabbae,* Mt. 9:32)
“They brought him a deafman.”
(20) Prinesi *darŭ*.                    (OCS*:Liber Sabbae,* Mt. 8:4)
carry gift.nom(/acc)
“Offer him the gift.”

In this way, learners started to be confronted with a consistent alternation between genitive and accusative cases in regular object position.

(ii) Other consistent alternations of a similar nature affected partial or partitive objects, quantified expressions, objects of negated verbs (the so-called genitive of negation), weak intensional verbs, cumulative verbs, and other similar prefixed quantificational verbs (with the prefixes *do*-, *za*-, *pri*-, *na*-; see Straková, 1961).

(21)    Iže      *ne*     vŭzĭmetŭ *kr(ŭ)sta* svoego.
who    not    takes            [cross        his].gen
(OCS: *Liber Sabbae,* Mt. 10:38)
“Whoever does not take up his cross…”

These types of bare genitive case are usually assumed to be licensed by some functional (rather than lexical) head and, therefore, “structurally determined” (Bailyn, 2004; Pereltsvaig, 2006; Kagan, 2013; etc.), thus giving rise to further genitive–accusative alternations in non-lexical positions.

#### Formalizing the change from genitive into accusative objects of active verbs

The change experienced by the active verbs reviewed so far can be formalized in the following way: initially, these verbs had the ability to take genitive objects, as depicted in (22), corresponding to (16a), repeated below:

(22)    Genitive lexical case pattern (the early Slavic pattern)
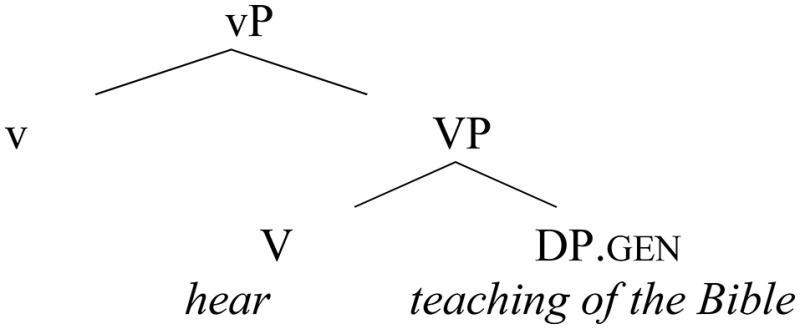
(16b)   I      *knižnago   poučcen*ь*ja*     slušaita.
           and  [bookish    teaching].gen listen
                                (Laurentian Chronicle, 151b)
           “And you both listen to the teaching ofthe Bible.”

Parallel changes that were taking place in the language at that time (tied to a general typological change in the language) affected mainly two structures: (i) bare genitive adjuncts being replaced by overt PPs (23), corresponding to (11a); and (ii) bare genitive objects alternating with accusative forms in non-lexical (structural) positions (24–25), representing (19b) and (21), respectively.

(23)    Replacement of bare genitive adjuncts by PPs
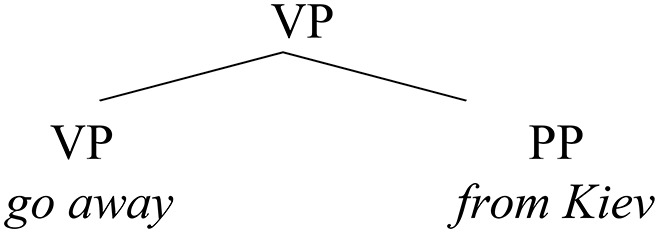
(11a)   Otstupi   voleju     *is*       Kyeva.
  left         by-will   from   Kiev.gen
  *(1^st^ Novgorod Chronicle—Commission roll, 112b)*
  “He moved away from Kiev of his own free will.”
(23)    Genitive animate NPs in regular object position
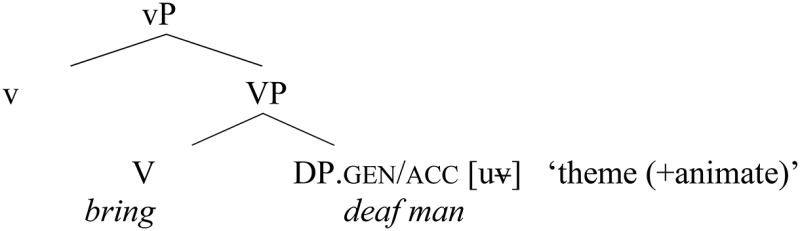
(19b)  Privedosę   emu  *č(e)l(ově)ka  gluxa.*
 carried        him   [person         deaf].gen(/acc)
                                                 (OCS: *Liber Sabbae,* Mt. 9:32)
           “They brought him a deaf man.”
(25)   Genitive and accusative NPs alternating in other nonlexical positions
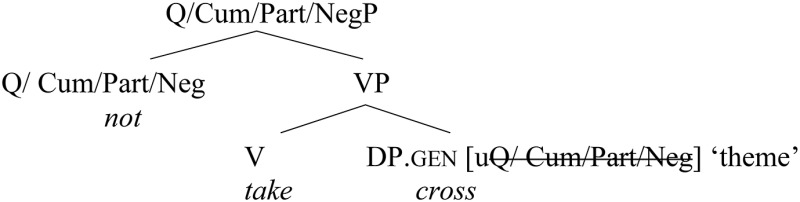
(21)  Iže    *ne*   vŭzĭmetŭ   *kr(ŭ)sta   svoego*.
         who  not   takes        [cross       his].gen
                                              (OCS: *Liber Sabbae,* Mt. 10:38)
         “Whoever does not take up his cross…”

Finally, the result of the change, the regular accusative object case pattern is represented in (26), corresponding to example (17):

(26)   Regular accusative object case pattern (the Middle Russian pattern)
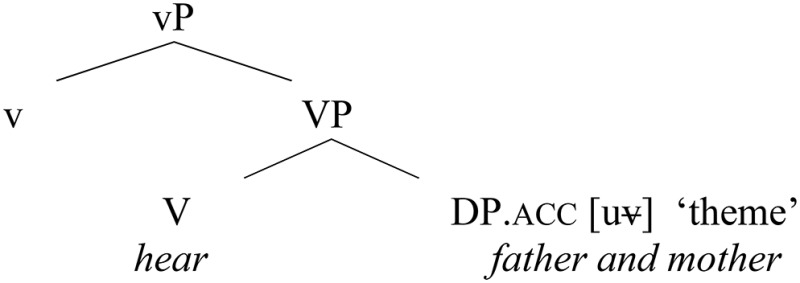
(17)   Ašče     kto   o(t)ca                  i      *m(a)t(e)rь*
if          who  father.gen/acc  and  mother.acc
ne poslušaetь.
not hears
“If somebody does not obey his father and mother.”

The change described here can be interpreted according to the concepts outlined in the introductory theoretical section. First, it arises because learners are confronted with innovative pieces of data as part of the Primary Linguistic Data (PLD) they receive, up to a point when their grammar stops converging with the one that generated the relevant input (“discontinuity of transmission between generations”). Language acquisition is therefore a fundamental piece in this process.

Further, as described in the introductory theoretical section, the contingency of grammar change underlies also this specific alternation of the Russian language, as it was determined by the unpredictable alteration of the conditions of the genitive—accusative alternation in other parts of Russian grammar (often related to non-syntactic factors, such as morphological syncretisms).

On the other hand, there seems to exist some bias operating in the previous changes reviewed in this section. There is little doubt that the set of changes associated with a global shift in the basic word order of the language, ultimately responsible for the replacement of bare lexical cases by PPs, is recurrent also in other Indo-European groups of languages (see references above), and seems to respond to some sort of economy or efficiency factor; in this case, to a general tendency to unify the head directionality of the language.

### Second step of the change: genitive into accusative complements of –*sja* verbs

#### The morphosyntactic development and formation of –*sja* verbs

The second phase in the loss of bare genitive case in Russian affected the verbs including a –*sja* suffix, plus the prefixed active verbs *izbegat'* “avoid” and *dostigat'* “reach,” to be treated as an exception in the final section. As introduced before, almost all the verbs of fear and avoidance (except *izbegat'* and *dostigat'*) are suffixed –*sja* forms (*bojat'sja* “to be afraid,” *storonit'sja* “to avoid,” *pugat'sja* “to be frightened,” *strašit'sja* “to dread,” *lišat'sja* “to be deprived,” etc.). We include in this section also the weak intensional or “potential” –*sja* verbs *slušat'sja* “to obey” and *dožidat'sja* “to expect” (the only non-active representatives in their group nowadays).

The history of the –*sja* verbal suffix in Russian is reviewed in detail in Zaliznjak (2008). This suffix has its origin in the clitic *sę*/*sja*, a free morpheme that was in fact the accusative form of the reflexive pronoun (cf. *se* in Romance languages). As such, it could be the object of any active verb regularly taking an accusative complement:

(27)   Na gorě eže     *sja*          nyne *zovet*ь         Ugorьskoje.
          on hill   which refl.acc  now call.3sg.active Ugorskoe
                                                     *(Laurentian Chronicle, 8)*
          “On the hill, which is now called Ugorskoe.”
          (cf. Spanish = ***se** llama*/Modern Russian:
          *nazyvaet**sja***.3sg.passive)

In Old Church Slavonic and Old Russian, unlike in other Indo-European languages (e.g. Ancient Greek), the accusative clitic *sę*/*sja* filled the internal argument position until the sixteenth century (Madariaga, 2010). In Old Russian, these elements could behave as a second-position clitic (28b), or a weak pronoun, usually following the verb (28a), but also following other elements, such as a preposition (Zaliznjak, 2008, p. 36). As we see in example (28b), when the first position in the sentence was occupied by a verb, the clitic could look much like a non-second-position -*sja* to learners (28a), because *sja*, in both patterns, followed the verb:

(28)   a.  Knjazja                  boi*sja.*
     prince.gen            fear.refl.
                                      *(Anthology of 1076,* 46)
     “Be afraid of the prince.”
    b.  Uboiši *sja*              *ot              lica*
     fear                       refl.           from
     sillьnaago.             *(Anthology of 1076, 141b)*
     [person strong].gen
              “You are afraid of a strong person.”

This kind of input could eventually lead learners to reinterpret the free *sja* as an element associated to a verb. Thus, the free morpheme merged in Old Russian with the verbal form, grammaticalizing later as a verbal suffix (Zaliznjak, 2008). As a final step of this morphological process, the –*sja* suffix underwent phonological reduction into just a palatalized -*s'* in certain environments (in regular conditions, after a final vowel):

(29)  *Imeni moego*        strašilisь.
         [name my].gen    feared
*(Tale of Yeruslan Lazarevich,* 330, in Borkovskij, 1978,
                                                                             p. 353)
“They were afraid of my name.”

Coming back to the list of the –*sja* verbs alternating between an accusative and genitive pattern in Russian nowadays, we can easily notice that virtually all of them are just the –*sja* “counterpart” of one of the active verbs of avoidance (30a) or potential verbs (30b) reviewed in the previous section. Even nowadays their morphological formation is fully transparent in most cases: the suffix –*sja*/-*s'* is just attached to the active form:

(30)   a.  *Lišat’* “deprive” > *lisat’sja* “be deprived”
              *Opasat’* “guard” > *opasat’sja* “mistrust”
              *Pugat’* “frighten” > *pugat’sja* “fear”
              *Strašit’* “frighten” > *strasit’sja* “fear”
              *Stranit’* “remove” > *storonit’sja* “avoid” etc.
    b.  *Slušat’* “listen” > *slušat’sja* “to obey”
    *Ždat’* “wait” > *dožidat’sja* “expect”      (special
                                formation with additional suffix)

Only the verb *bojat'sja* “to be afraid” (Old Church Slavonic *bojati sę*) did not correspond to an active verb as such, although prehistoric stages of the language probably displayed an active equivalent as well. Its active counterpart can be traced back to the proto-Slavic form ^*^bojati, not attested as such in historical Slavic, but related to equivalent Sanskrit or Baltic forms. All other verbs of avoidance (30a) display from Middle Russian an active form taking an accusative object, and a –*sja* form taking a genitive object (the one that has recently start to alternate with accusative).

As for the forms in (30b), at the beginning of the twentieth century, by Peškovskij's (2001 [1938]) time, they had two interesting properties: (i) they were the only members of their lexico-semantic families preserving genitive case (not having changed into an accusative pattern or an alternating pattern determined by the semantics of the object), and (ii) they were the only members of their families with the –*sja* suffix.

Now, after having surveyed all the relevant data, I will propose a formalization of this shift and explain why this change is still taking place nowadays, whereas their active counterparts changed four centuries ago.

#### Formalizing the change from genitive into accusative objects of –*sja* verbs

The original pattern included a free pronominal *sja* element in object position, which could behave as a second-position clitic, as in Old Church Slavonic (cf. example 27). The “avoided” element (i.e., the element causing fear) was associated with the semantics of separation, and marked with genitive (<ablative) case or an overt PP, as usual in most early Indo-European languages. This is illustrated in (31), representing (28b):

(31)   Old pattern with a free accusative *sja* clitic
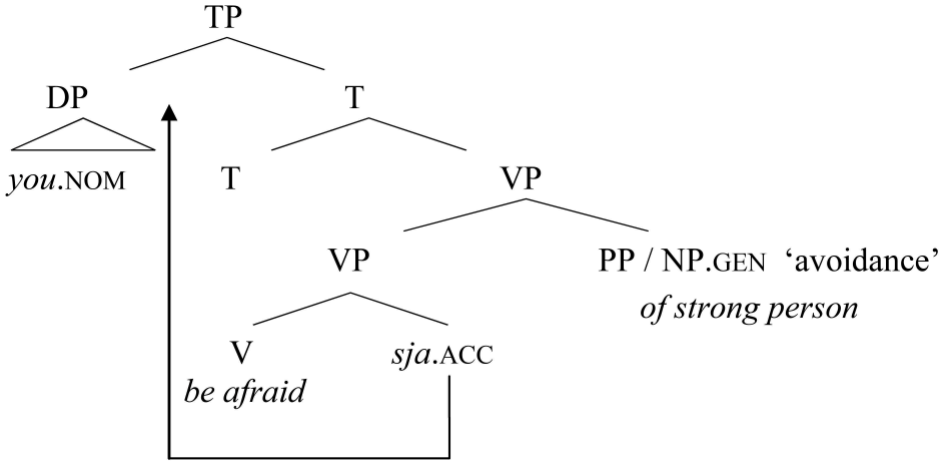
(28b) Uboiši   *sja    (ot)      lica silьnaago.*
fear       refl.  (from)  [person strong].gen
                              *(Anthology of 1076, 141b)*
         “You are afraid of a strong person.”

In Old Russian, the free morpheme *sja* had the possibility of staying in a lower position and being attached to the verb. This initial short movement prior to reanalysis is represented in (32), corresponding to (28a), in which the pronoun *sja* does not behave as a second-position clitic, but surfaces attached to the verb.

(32)   First morphological incorporation of −*sja* (merge and move)
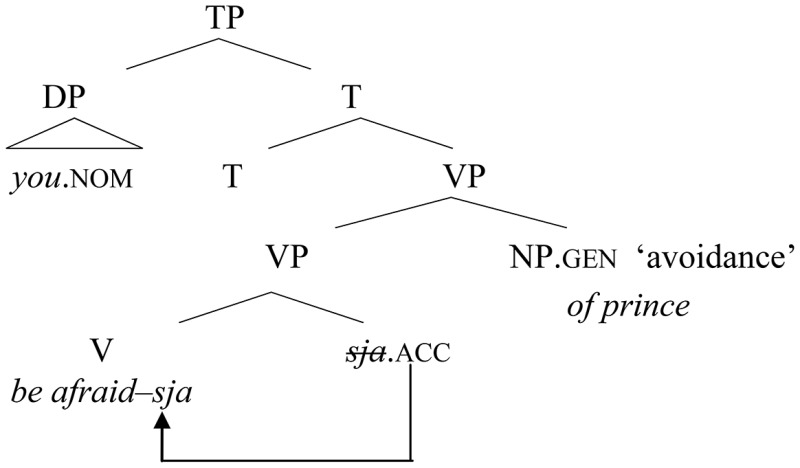
(28a)  Knjazja        *boisja.      (*Anthology of 1076,* 46)*
           prince.gen  fear.refl.
          “Be afraid ofthe prince.”

By this time, the complement slot was still occupied by an overt element, which, by virtue of Burzio's (1986) Generalization, banned accusative assignment to any other possible object. The incorporation of *sja* into the verbal form, which represents the initial step of the change under study here, was completed in Middle Russian (Zaliznjak, 2008, p. 217ff), but it did not automatically convey any further change in the structure at this stage.

Later on, the *sja* element lost its ability to behave as a clitic, and became fully incorporated into the verb in a very common diachronic process classically known as grammaticalization. These kinds of processes have been described in generative accounts as up-the-tree movements, followed by the reanalysis of the element initially moved as base-generated in the landing position (see Roberts and Roussou, 2003). Again, this grammaticalization process was a necessary previous step for later reanalysis, but still did not involve any major change in case assignment. The verbs affected were still acquired as “exceptional” in that their complement was marked with quirky genitive case.

(33) Reanalysis of −*sja* as directly merged in V
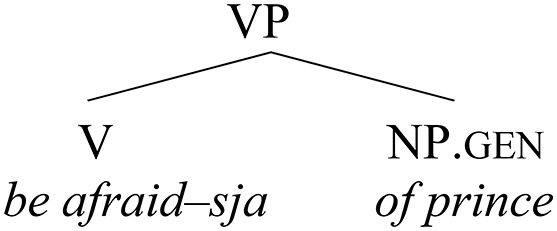

In some speakers, however, at some point in the recent history of Russian, after the morpheme –*sja* started to be base-generated in V, the whole element in this position could start to be perceived as a “deponent” verb. In other words, the complement slot became free, and the historically “disturbing” bare quirky genitive could finally be reanalyzed as a regular accusative object, merged as a complement of the verb. This is depicted in (34), corresponding to (2a), repeated below:

(34) Reanalysis of the verbal complement
        (> shift in case assignment)
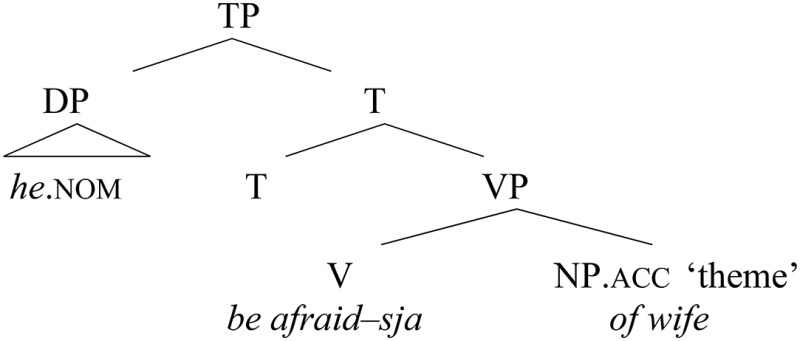
(2a) On         boitsja *ženu*.
        he.nom fear     wife.acc
        “He is afraid of his wife.”

The nature of the shift represented in these structures evidences the fact that the ultimate reason for the alternating case patterns with –*sja* verbs, changing recently in Russian, was in fact the reorganization of bare lexical cases four centuries before. In Middle Russian, active verbs taking a genitive complement changed into an accusative pattern (or a semantically determined alternating pattern in the case of weak intensional verbs). But –*sja* verbs behaved in a different way. They preserved a quirky genitive complement longer because at the crucial moment of the reorganization of the bare case system in Russian, they still fell under Burzio's Generalization; i.e., the complement slot was still filled by the element *sja*, and learners were not able to replace the “disturbing” genitive NP with an accusative NP, as they did in the case of active verbs.

Learners did not have any other option than acquiring the quirky pattern as “exceptional,” as it is still acquired nowadays, i.e., by means of some special morphological rule assigning genitive case to the relevant objects at the Externalization component of the language (Sigurðsson, 2012). This is also in line with the theories about competing grammars coexisting in a single speaker at different linguistic levels, as stated in the introductory theoretical section.

However, after the whole verbal form was reanalyzed as a unique element merged in V, freeing up the complement slot, reanalysis of the “avoided” element as a regular accusative object became available, which is in fact what eventually happened in colloquial language. Again, as explained in the introductory theoretical section, contingent unpredictable conditions determine here the possibility for a syntactic phenomenon to undergo change in a regular way, or the need to “wait” until something else happens in the language, making the conditions for change favorable. The case addressed in this paper is of special interest, because it also confirms the idea that variation can correspond to successive discrete changes spreading further to new syntactic environments.

#### On gradualness and discreteness of change

As said before, the seemingly gradualness of a change in progress can often correspond to a diversity of linguistic environments successively affected by one unique change. Here too, what seems as a gradual change can be reduced to a series of discrete changes affecting different items or structures at different moments, according to a “third factor” effect, namely, Input Generalization (“maximize available features”); see the introductory theoretical section. If change spreading proceeds in this particular way, we expect the presence of different “splits” between competing variants according to different features, structures, or lexical items. These considerations also apply in our case study.

The major split between the alternating patterns at issue was between active and –*sja* verbs, which have been shown to feature a very clear structural contrast (the availability or not of a free complement slot in the structure).

But other minor splits in this process must also be taken into account, namely, those determined by (in)animacy and declension classes^8^. Some speakers favor accusative case only when the object of the verb *bojat'sja* conveys an animate feature (cf. footnote 2); others reject accusative assignment when the object belongs to III declension class, even if it is animate. These patterns are illustrated in (35):

(35) Ja  bojus’ mamu           /^*^ grozu         /^*^ grom
I   fear     Mum.acc.ii /storm.acc.ii  /thunder.acc.i
/^*^ mat’.
/mother.acc.iii

Splitting alternating patterns according to some specific semantic feature or morphological class is a recurrent way of pinpointing a change process. In the specific case of Russian, animacy and declension class have played this role before: animacy determine the case patterns for masculine singular I class, and all plural objects (see example 6).

Other speakers, however, have gone further in this process and are able to use accusative case almost regardless of the animacy feature of the object, whether of class I or II (36a-b). Some are tolerant with III declension class objects, too (37a-b)^9^.

(36) a.  U           nas           inogda         daže
 at           us             sometimes  even
 bojatsja* bumagu*.  (Alešin*, Vstre*c*i na grešnoj zemle)*
 fear       paper.acc.ii
“In our  country, they are sometimes afraid of some
piece of paper.”
(Google search,
             https://otvet.mail.ru/question/92115035)
b. A      èlektriki           bojatsja    *grom*                i
and  electricians      fear          thunder.acc.I and
A      èlektriki           bojatsja *   grom*               i
molniju?
lightning.acc.ii
“Are electricians afraid of thunder and lightningč”
(39) a. Ja           bojus’           svoju         *mat*’.
 I           fear             own       mother.acc.iii
(30 times in a Google search)^10^
b. Razve     možno    bojat’sja     *myš’?*
maybe   possible  fear            mouse.acc.iii
—udivilsja Birjukov.
surprised Biriukov
(Petkevic, *Živye cvety zimoj*)
“Is it possible to be afraid of a mouse?
-asked Biriukov with surprise.”

A final observation on splits in the alternating patterns concerns the spread of the new accusative form according to different lexical items, thus rendering again a succession of discrete changes, as explained in the introductory theoretical section of this paper. As expected, more frequent verbs are more prone to be used with accusative case than others. As noted by Nesset and Kuznetsova (2015a), there are differences between them even in the case of frequently used verbs; namely, *slušat'sja* and *dožidat'sja* are changing faster than *bojat'sja*. This correlation is perhaps not random: as shown in (30b) above, both *slušat'sja* and *dožidat'sja* have active counterparts, which had changed into an accusative pattern earlier, while *bojat'sja* never had one. This fact suggests that *bojat'sja* was maybe less prone to Input Generalization, and therefore more resistant to change, regardless of its frequent use in the language.

#### The exceptions: the verbs *dostigat*' “reach” and *izbegat*' “avoid”

To conclude this section, let us now recall the only active verbs that exceptionally preserved bare genitive objects in the Russian language: *dostigat'* and *izbegat'*. Although they are active, these verbs behave as –*sja* verbs in the sense that they started to change later, and nowadays display, in principle, the alternation addressed in this paper, as shown in (40):

(40) a.  Izbegat’ * ètoj vetki*                /% *ètu vetku*
 avoid      [this branch].gen/   [this branch].acc
metro.
subway
“Avoid this branch ofthe subway.”
 b. Dostigat’ *svoej celi*                       /% *svoju cel’.*
reach     [own objective].gen/      [own objective].acc
“Reach one’s own objective.”

These two verbs are prefixed forms in their basic form, unlike the other active verbs that changed early in the language from genitive into accusative case. On the other hand, they are the only ones lacking a -*sja* counterpart; in other words, like *bojat'sja* (this one lacking an active counterpart), they did not enter the alternation active–medial illustrated in (30) above.

Their special morphology could probably help them to preserve bare genitive case marking as not so “disturbing” to be acquired by learners. In fact, we have independent evidence that bare genitive case was preserved until late Middle Russian, when it was associated with prefixed verbs (Černyx, 1952). The examples in (41) include a verbal prefix, called preverb in the indoeuropeanist tradition, which is identical to the “missing” preposition, making it easily recoverable^11^.

(41) a.  I     tu *    na*ěxali *nas       *tri     tatariny     poganye.
             and here flung    us.gen three Tartars      evil
            (Afanasi Nikitin’s Journey, 19)
            “And then three evil Tartars attacked us.”
           (Later: [_PP_ *na* nas] “toward us”)
b. Da   sluxъ * nasъ*    totъ  došelъ.
     and rumor us.gen this   came
*            (Historical acts* 2, 333, in Černyx, 1952, p. 270)
    “This rumor has come to us.”
    (Later: [_PP_ *do* nas] “to us”)

Likewise, the prefixes in *dostigat'* “reach” and *izbegat'* “avoid,” as well as the lack of voice alternation, could contribute to the longer preservation of the bare genitive complement of the verbs. Again, this development is in line with the introductory notions about successive realizations of one unique change in different morpho-syntactic conditions (see the introductory theoretical section).

## Conclusion

In this paper, I have shown the convenience of introducing diachronic analyses into the study of synchronic syntactic phenomena through the practical example of a case alternation in Modern Russian: accusative objects (colloquial pattern) vs. genitive objects (neutral pattern) of the –*sja* verbs denoting avoidance, and the verbs *slušat'sja* “obey” and *dožidat'sja* “expect.”

First, I have reviewed the virtues and shortcomings of previous non-formal accounts about this phenomenon, as well as the potential application of a formal synchronic account to this phenomenon.

Then, I have shown that a formal account including the diachronic dimension is more explanatory. In what sense? The diachronic analysis allows us to realize that the alternation in case marking associated nowadays to –*sja* verbs does not just correspond to a set of morphological rules, but has an additional underlying syntactic explanation. These verbs are now undergoing the same change from genitive into accusative case marking that their active counterparts underwent several centuries ago. This change was ultimately tied to the general typological shift experienced in early Slavic, which led to the reorganization of bare lexical cases, especially bare genitive case. The reason why –*sja* verbs started to change later than active verbs is also syntactic: until the sixteenth century, *sja* was an accusative free morpheme, merged as the complement of V; this prevented the change from genitive into accusative marking, which was taking place in active verbs by that time. Merging *sja* with the verb eliminated the obstacle for accusative marking and opened the possibility for these verbs to change following the same path active verbs had undergone some centuries before.

The rest of features characterizing the distribution of the variants according to animacy, declension classes, and lexical items are also accounted for with the help of the diachronic data: splitting the available variants in successive discrete changes according to semantic features (animacy) or morphological features (declension class) is a recurrent phenomenon of pinpointing diachronic processes. On the other hand, a higher difficulty in applying third-factor strategies (Input Generalization) to certain lexical items suggests that these items will be less prone to change, as happens with less frequently used verbs, and also with the verb *bojat'sja* (compared to *slušat'sja* and *dožidat'sja*), because it lacks an active counterpart taking an accusative object. Likewise, differential morphology was probably the reason for slowing down the expected development of the only active verbs (*dostigat'* and *izbegat'*) that preserved the type of case alternation at issue even nowadays.

## Author contributions

The author confirms being the sole contributor of this work and approved it for publication.

### Conflict of interest statement

The author declares that the research was conducted in the absence of any commercial or financial relationships that could be construed as a potential conflict of interest. The reviewer RE and handling Editor declared their shared affiliation, and the handling Editor states that the process nevertheless met the standards of a fair and objective review.

## Footnotes

^1^In the last two groups (types ii and iii), there are two active verbs (without the prefix –*sja*) that display in Modern Russian the alternating genitive vs. accusative pattern addressed in this paper, as in examples (1–2): *izbegat'* “avoid” (type ii), and *dostigat'* “reach” (type iii). We will come back to them later on.

^2^Even if some Russian speakers seem to display a semantically-oriented pattern in the distribution of case marking in the verbs of avoidance (at least, with the verb *bojat'sja*), this distribution corresponds to a different semantic feature, namely, the (in)animacy of the NP object (Ora Matushansky p.c.), and not an unreal vs. real feature, as in active weak intensional verbs, as we will see later on.

^3^On the one hand, Russian children are taught at school that the “correct” case form for these objects is genitive (at least from Peškovskij, 1918, p. 78). On the other, the role of linguistic priming becomes evident when confronting speakers with a specific language chunk to which they have been frequently exposed. In this case, they will choose the case marking variant corresponding to the “familiar” chunk, even if the alternative form is also in principle available. For example, when speakers are asked to complete the sentence *My boimsja volka i ___* “we are afraid of the wolf and ___”, by choosing between the form *sovy*.GEN and *sovu*.ACC “owl,” they unanimously choose the second variant. This is because the accusative variant is precisely the one heard in a very similar sentence of a famous song in the film *Brilliantovaja ruka* (“Diamond arm”).

^4^Accusative object case (as opposed to genitive case) is widely acknowledged to be the colloquial variant in the relevant case alternation, and this Google search is provided just as an illustrative example of this fact. After searching for the relevant combinations with the word order “V + complement” (restricting the search to Russia), cleaning up the hits obtained after each search, and discarding repeated and irrelevant results, we obtain the following figures: the “expected” combination of a colloquial lexical item and accusative case marking (*mamu*) renders 123 occurrences, while *bojat'sja mamy* (colloquial lexical item, genitive case marking) renders 86 occurrences (58.8 and 41.2%, respectively). With the more formal lexical item *mat'*, both case marking options diverge much more: *bojat'sja mat'*—41 occurrences vs. *bojat'sja materi*—124 occurrences (25 and 75%, respectively).

^5^An illustrative example of this development is the parallel loss of the early IE absolute participial constructions in the different IE groups (*ablativus absolutus* in Latin, absolute genitive in Greek, absolute dative in Slavic, etc.), and their later replacement by different circumstantial complements headed by an overt preposition or conjunction (Bauer, 1995).

^6^The *Commission roll* is a fifteenth-century copy of the *1*^*st*^ *Novgorod Chronicle* (thirteen to fourteenth century) and *Radziwill Chronicle* is a late fifteenth-century copy of the *Laurentian Chronicle* (fourteenth century).

^7^In Old Church Slavonic, bare genitive alternated with a PP *ot* “from” + genitive case (Borkovskij, 1978, p. 353), a pattern occasionally found in Russian as an archaism until the seventeenth century:

^8^Class I refers to the 1st declension class (-*o* stems); class II is the second declension class (-*a* stems), and class III stands for the third declension class (-*i* stems).

^9^The verbs most frequently combined with accusative case (*slušat'sja* and *dožidat'sja*) accept inanimate and III declension class objects (*slušat'sja mat'/Minfin* “obey mother.acc.iii/Ministry of finances.acc.i”) more often than *bojat'sja*.

^10^After a search of the exact phrase in (39a), only in Russian pages, I cleaned up the irrelevant and repeated hits, and obtained 30 different occurrences of it. This shows that the use of accusative case with 3rd declension class is not rare at all in the language.

^11^Preverbs of this kind are found in other early Indo-European languages, most famously in Homeric Greek, where some of these adverbial elements were multifunctional; i.e., the same element could behave as a free adverb, a preposition, a postposition or a preverb correlating with a bare lexical case-marked NP, as in the Russian examples in (41).
